# PSPH Promotes Hepatocellular Carcinoma Progression by Upregulating HIF-1α and Is Regulated by LncRNA GSEC/miR-101-3p

**DOI:** 10.3390/cimb48080784

**Published:** 2026-07-31

**Authors:** Yi Yang, Hang Min, Yuanting Huang, Lingjing Tao, Hao Zhou, Wenbo Zhang, Xiaoshuai Ren, Changyun Huang, Yang Deng, Jun Zhou

**Affiliations:** College of Life Sciences and Health, Wuhan University of Science and Technology, No. 2 Huangjiahu West Road, Hongshan District, Wuhan 430081, China; 1843966176@wust.edu.cn (Y.Y.); 18895674063@163.com (H.M.); 18744824617@163.com (Y.H.); peach1736449631@wust.edu.cn (L.T.); 15629587779@163.com (H.Z.); z517007089@163.com (W.Z.); 18737731057@163.com (X.R.); 18202797377@163.com (C.H.)

**Keywords:** HCC, PSPH, angiogenesis, HIF1α

## Abstract

Hepatocellular carcinoma (HCC) lacks effective early diagnostic markers and is heavily driven by angiogenesis and hypoxia. Emerging evidence indicates that metabolic enzymes and noncoding RNAs coordinate these processes. Here, we reveal that phosphoserine phosphatase (PSPH), a serine biosynthesis enzyme, is significantly upregulated in HCC, correlating with angiogenic markers and poor prognosis. Clinical data and functional assays demonstrated that miR-101-3p directly targets the PSPH 3′ UTR to suppress its expression, while lncRNA GSEC acts as a competing endogenous RNA to sponge miR-101-3p. In vitro, GSEC knockdown or miR-101-3p overexpression decreased PSPH and HIF1α levels, strongly inhibiting HCC angiogenesis, migration, invasion, and proliferation. Crucially, these anti-tumor effects were reversed by restoring PSPH. In vivo, modulating the GSEC/PSPH axis significantly altered xenograft tumor growth and vascularization. Conclusively, the GSEC/miR-101-3p/PSPH regulatory axis drives HIF1α-dependent angiogenesis and HCC progression, highlighting PSPH as a promising diagnostic biomarker and therapeutic target.

## 1. Introduction

Hepatocellular carcinoma (HCC) remains a major cause of cancer-related mortality worldwide and accounts for the majority of primary liver cancers, with a substantial global death toll each year [1,2]. Because early-stage HCC is frequently asymptomatic and reliable early diagnostic methods are lacking, many patients present with advanced disease and receive palliative systemic therapies that confer only modest survival benefits [3,4,5,6]. Therefore, an improved understanding of molecular drivers and the identification of robust biomarkers are critical to enable earlier detection and to expand therapeutic options for HCC patients [7,8,9].

Tumor hypoxia and hypoxia-inducible factor 1 alpha (HIF1α) signaling are central to HCC progression, promoting angiogenesis, metabolic reprogramming, immune evasion, and therapeutic resistance [10,11]. Angiogenesis, in particular, is a hallmark of HCC and underlies both tumor growth and metastatic dissemination; consequently, factors that couple metabolic adaptation to hypoxia-driven vascular remodeling are of high translational interest. Metabolic enzymes that influence intracellular redox state, one-carbon metabolism, or amino acid availability can modulate hypoxic responses and the tumor immune microenvironment, thereby affecting angiogenic programs. However, the specific links between serine metabolism and HIF1α-dependent angiogenesis in HCC remain incompletely defined [12].

Phosphoserine phosphatase (PSPH) catalyzes the terminal step of the de novo serine biosynthesis pathway and has been implicated in cancer cell proliferation, metabolic adaptation, autophagy, and immune modulation across multiple tumor types [13,14,15,16]. PSPH overexpression has been reported in diverse malignancies and has been associated with tumor progression and poor prognosis [17,18,19,20,21]. Recent studies also suggest reciprocal interactions between metabolic enzymes and hypoxia signaling that may form feed-forward loops in the tumor microenvironment [22,23,24,25,26,27,28,29,30,31,32,33,34,35,36]. Nevertheless, the contribution of PSPH to HCC metastasis, angiogenesis, and potential immune evasion has not been systematically characterized, and the upstream regulatory mechanisms that control PSPH expression in HCC are poorly understood.

Noncoding RNAs, including microRNAs (miRNAs) and long noncoding RNAs (lncRNAs), are established post-transcriptional regulators of oncogenic networks and can act as critical modulators of metabolic and hypoxia-responsive pathways [37,38,39,40,41,42,43,44,45,46,47,48]. LncRNAs may function as competing endogenous RNAs (ceRNAs) to sequester miRNAs and thereby derepress target mRNAs, a mechanism increasingly recognized in HCC biology [49,50,51,52]. In particular, miR-101-3p and lncRNA GSEC have been implicated in cancer-related processes, but their roles in regulating PSPH and HIF1α-dependent angiogenesis in HCC remain to be defined.

Although PSPH has been reported to participate in metabolic adaptation and tumor progression in several malignancies, its role in HIF1α-dependent angiogenesis in hepatocellular carcinoma has not been elucidated. Moreover, no previous studies have identified the GSEC/miR-101-3p/PSPH axis as a regulatory pathway in HCC, and the upstream non-coding RNA network controlling PSPH expression remains largely unknown. Therefore, our study fills this important knowledge gap by demonstrating that GSEC sponges miR-101-3p to derepress PSPH, which in turn enhances HIF1α signaling and promotes angiogenesis and HCC progression.

In the present study, integrative bioinformatic analyses and experimental validation were employed to investigate whether PSPH contributes to HCC metastasis and angiogenesis via HIF1α-related mechanisms and to determine whether PSPH expression is regulated by a lncRNA/miRNA axis. Specifically, TCGA/GTEx data mining, luciferase reporter assays, and in vitro and in vivo gain- and loss-of-function experiments were used to define the regulatory relationship among lncRNA GSEC, miR-101-3p, and PSPH. Ultimately, our findings reveal that the GSEC/miR-101-3p/PSPH axis promotes HIF1α-dependent angiogenesis and HCC progression, highlighting PSPH as a promising diagnostic and therapeutic target.

## 2. Materials and Methods

### 2.1. Bioinformatics Analysis

To ensure transparency and reproducibility, all bioinformatics analyses were performed using clearly defined databases, analytical thresholds, and gene selection criteria. In this manuscript, “HCC” refers to hepatocellular carcinoma as a disease, whereas “LIHC” specifically refers to the TCGA-LIHC dataset used for expression profiling and clinical information. Expression profiles and clinical information for liver hepatocellular carcinoma (LIHC) were obtained from the TCGA LIHC dataset.

The XianTao platform (https://www.xiantaozi.com/), based on TCGA LIHC data, was used to analyze the expression levels of PSPH, miR-101-3p, and GSEC, as well as to perform patient survival analysis, Cox proportional hazards regression, and immune cell infiltration assessment. Differentially expressed genes (DEGs) were defined using the thresholds adjusted *p* < 0.05 and |log_2_FC| > 1.

The StarBase database (https://rnasysu.com/encori/, (accessed on 15 March 2023)) was used to evaluate the correlation between PSPH and hsa-miR-101-3p, and to predict the putative binding sites between hsa-miR-101-3p and lncRNA GSEC. Co-expression gene clustering for PSPH was performed using LinkedOmics, followed by GO and KEGG enrichment analyses via the DAVID database (https://ngdc.cncb.ac.cn/databasecommons/database/id/3061 (accessed on 15 March 2023)).

Correlations between PSPH and markers of the VEGF signaling pathway were assessed using GEPIA2 (http://gepia2.cancer-pku.cn/). Potential miRNAs targeting PSPH were predicted using DIANA, StarBase, mirDIP, and miRmap, and the intersecting candidates were integrated and visualized using FunRich.

### 2.2. Cell Culture

Human umbilical vein endothelial cells (HUVECs) and HEK293T cell lines were obtained from the BeNa Culture Collection (Beijing, China). The normal human hepatic cell line LO2 and the HCC cell line HepG2 were purchased from Pricella Biotechnology (Wuhan, China). The HCC cell line SMMC-7721 was obtained from Warner Bio (Wuhan, China). HUVECs, HEK293T, and HepG2 cells were cultured in Dulbecco’s Modified Eagle Medium (DMEM), while LO2 and SMMC-7721 cells were cultured in RPMI-1640 medium. All culture media were supplemented with 10% fetal bovine serum (FBS). HepG2, SMMC-7721, LO2, HEK293T, and HUVEC cells were cultured in DMEM, MEM, or RPMI-1640 supplemented with 10% fetal bovine serum (FBS) at 37 °C in a humidified incubator containing 5% CO_2_. Cells were routinely passaged at 70–80% confluence [53].

### 2.3. Plasmid Construction

For overexpression studies, the coding sequences of PSPH and lncRNA GSEC were cloned into the pLVX vector, while miR-101-3p was overexpressed using the pLKO.1 vector. For luciferase reporter assays, the wild-type (WT) and mutant (MUT) 3′UTR fragments of PSPH were inserted into the pGL3-promoter vector, and the full-length GSEC sequence was cloned into the pmirGLO vector. All cloning primers used in this study are listed in Supplementary Table S1.

### 2.4. Cell Transfection

Cells were seeded at 40–50% confluence and transfected using Lipofectamine 2000 (Thermo Fisher Scientific, San Diego, CA, USA) according to the manufacturer’s protocol. miR-101-3p mimics (50 nM), inhibitors (100 nM), or corresponding negative controls were added to the culture medium. For plasmid transfection, 1–2 μg plasmid DNA was used per well of a 6-well plate. Cells were harvested 24–48 h after transfection for RNA and protein analysis. Stable cell lines were generated by lentiviral infection at MOI = 10 in the presence of 8 μg/mL polybrene, followed by puromycin (2 μg/mL) or G418 (500 μg/mL) selection for 5–7 days [53].

### 2.5. Quantitative Real-Time PCR (qRT-PCR) Assay

Total RNA was extracted using TRIzol reagent (Invitrogen, Carlsbad, CA, USA) according to the manufacturer’s instructions. Reverse transcription was performed using a commercial reverse-transcription kit (Vazyme, Nanjing, China) to synthesize cDNA. For miRNA detection, the Hairpin-it miRNA qRT-PCR kit (Vazyme, Nanjing, China) was used. qRT-PCR was conducted on a Bio-Rad CFX96 Real-Time PCR Detection System (Bio-Rad Laboratories, Hercules, CA, USA) using 2 × SYBR Green qPCR Master Mix (Yeason, Shanghai, China). U6 served as the internal control for miR-101-3p, while GAPDH was used as the internal reference for mRNA quantification. Relative gene expression levels were calculated using the 2^−^ΔΔCt method. All primers were synthesized by TSINGKE (Beijing, China) [53]. All primers are shown in Supplementary Table S2.

### 2.6. Western Blot

Western blot analyses were performed as previously described [53]. The primary antibodies used in this study included: anti-PSPH (14513-1-AP, Proteintech, Wuhan, China), anti-HIF1α (20960–1-AP, Proteintech), anti-VEGFA (19003–1-AP, Proteintech), anti-VEGFR2 (26415–1-AP, Proteintech), and anti-β-actin (AC026, ABclonal, Wuhan, China). Horseradish peroxidase (HRP)-conjugated goat anti-mouse IgG (H + L) (AS003, ABclonal) and goat anti-rabbit IgG (H + L) (AS014, ABclonal) were used as secondary antibodies.

### 2.7. Wound Healing Assay

Cells were seeded into 6-well plates and grown to full confluence. A straight scratch was created across the cell monolayer using a sterile 200-μL pipette tip. Detached cells were removed by washing with PBS, and serum-free medium was added to eliminate proliferation-related effects. Images of the wound area were captured at 0 h and 24 h using an inverted microscope (Olympus, Tokyo, Japan). The wound closure rate was quantified using ImageJ software (version 1.50i, National Institutes of Health, Bethesda, MD, USA) [53].

### 2.8. Transwell

Cells were resuspended in serum-free medium, and 5 × 10^4^ cells were seeded into the upper chamber of a Transwell insert (8-μm pore size; Corning, NY, USA). For invasion assays, the upper chamber was pre-coated with Matrigel (BD Biosciences, San Jose, CA, USA) and allowed to polymerize at 37 °C. Medium containing 10% fetal bovine serum (FBS) was added to the lower chamber as a chemoattractant. After incubation for 24 h, non-migrated or non-invaded cells on the upper surface were removed with a cotton swab. Cells that had migrated or invaded to the lower surface were fixed with 4% paraformaldehyde, stained with 0.1% crystal violet, and counted under an inverted microscope (Olympus, Tokyo, Japan).

### 2.9. Clone Formation

Cells were digested into single-cell suspensions and counted using a hemocytometer. A total of 500–800 cells were seeded into each well of a 6-well plate and cultured in complete medium for 10–14 days until visible colonies formed. The medium was replaced every 3 days. At the endpoint, colonies were gently washed with PBS, fixed with 4% paraformaldehyde for 20 min, and stained with 0.1% crystal violet for 20 min. Excess dye was removed by rinsing under running water, and plates were air-dried. Colonies containing more than 50 cells were counted under an inverted microscope (Olympus, Tokyo, Japan) [54].

### 2.10. Tube Formation

To evaluate in vitro angiogenesis, cells from both the experimental and control groups were cultured, and the conditioned media (supernatants) were collected. Cellular debris and impurities were removed by passing the supernatants through a 0.45 μm filter. Concurrently, 96-well plates were coated with Matrigel and incubated at 37 °C until solidification. HUVEC suspensions mixed with the respective conditioned media were then seeded into the Matrigel-coated wells. The plates were incubated at 37 °C for over 3 h to allow for capillary-like tube formation. The vascular structures were subsequently observed and photographed using an inverted microscope.

### 2.11. Animal Model

Female BALB/c nude mice (3–4 weeks old) were maintained under specific pathogen-free (SPF) conditions with controlled temperature and humidity. For subcutaneous xenograft assays, tumor cells from each experimental group were resuspended in PBS, and 0.2 mL of cell suspension containing 2 × 10^7^ cells was injected subcutaneously into the left flank of each mouse. Mice were randomly assigned to the corresponding groups according to the injected cell type.

Tumor growth was monitored once per week using a digital caliper. Tumor volume was calculated using the formula: V = 0.5 × length × width^2^. After 28 days, mice were euthanized, and tumors were excised, photographed, and weighed. All animal procedures were approved by the Animal Ethics Committee of Wuhan University of Science and Technology and conducted in accordance with institutional guidelines [53]. The animal experiment procedure has been approved by the Laboratory Animal Center of Wuhan University of Science and Technology and the Experimental Animal Ethics Review Committee.

### 2.12. Statistical Analysis

To determine statistical significance, Student’s *t*-test or one-way analysis of variance (ANOVA) were utilized. * *p* < 0.05 was regarded as statistically significant [53].

## 3. Results

### 3.1. PSPH Overexpression in Hepatocellular Carcinoma Correlates with Angiogenic Markers

**Figure 1:**
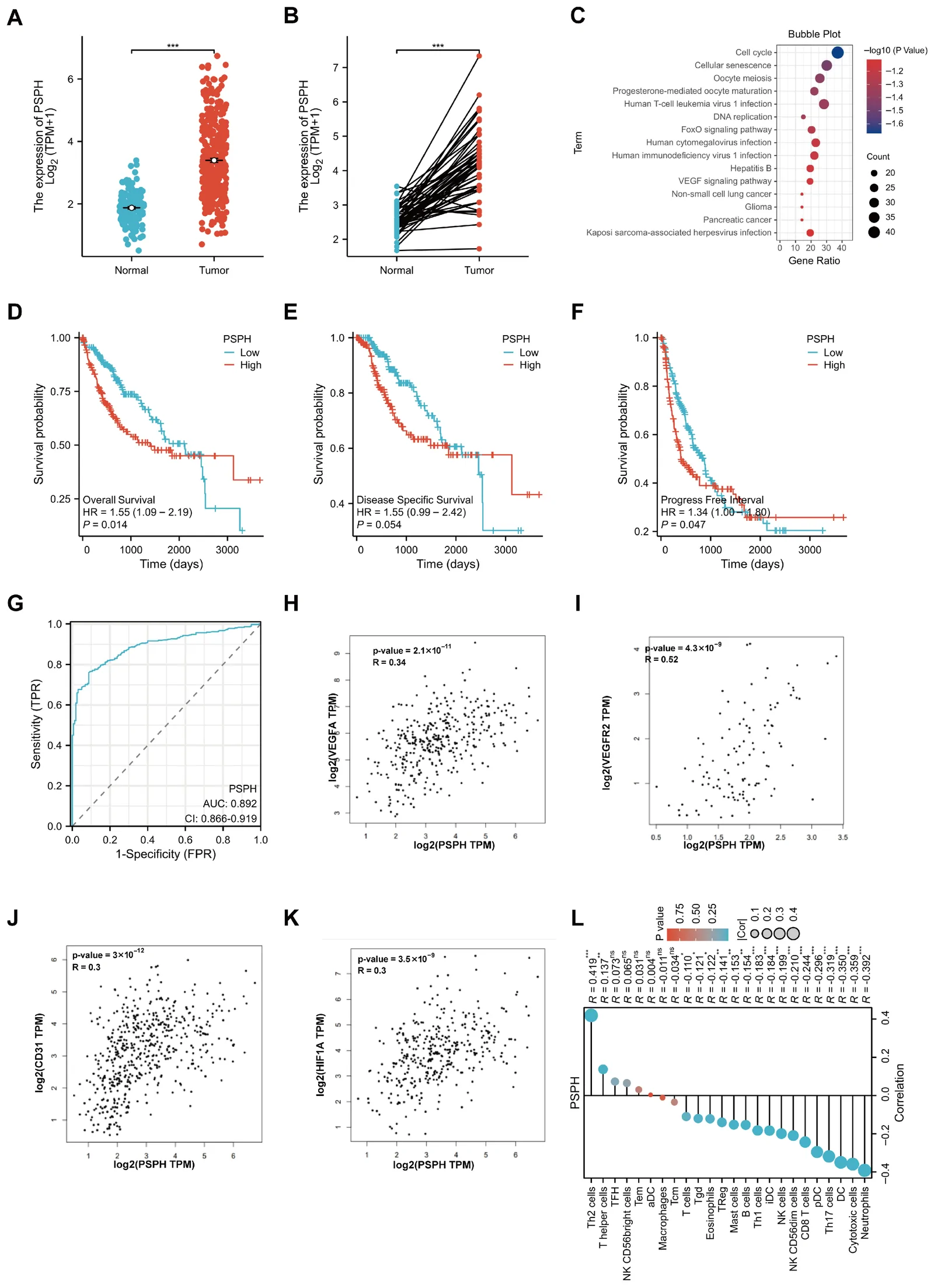
**PSPH is overexpressed in hepatocellular carcinoma (HCC) samples and correlates with angiogenesis markers and immune infiltration.** (**A**) The mRNA expression levels of PSPH in normal and HCC (LIHC) tissues from the TCGA and GTEx databases; (**B**) The mRNA expression levels of PSPH in paired tumor and adjacent normal tissues from TCGA-LIHC patients; (**C**) Enrichment analysis of PSPH-associated differentially expressed genes presented as a bubble plot; (**D**) Kaplan–Meier survival curve showing the association between PSPH expression and overall survival (OS) in HCC patients; (**E**) Kaplan–Meier survival curve showing the association between PSPH expression and disease-specific survival (DSS) in HCC patients; (**F**) Kaplan–Meier survival curve showing the association between PSPH expression and progression-free interval (PFI) in HCC patients; (**G**) Receiver operating characteristic (ROC) curve evaluating the diagnostic value of PSPH in HCC; (**H**) Correlation between the expression levels of PSPH and VEGFA in HCC; (**I**) Correlation between the expression levels of PSPH and VEGFR2 in HCC; (**J**) Correlation between the expression levels of PSPH and CD31 in HCC; (**K**) Correlation between the expression levels of PSPH and HIF1α in HCC; (**L**) Correlation between PSPH expression and the infiltration levels of various immune cell populations in the tumor microenvironment. * *p* < 0.05, ** *p* < 0.01, *** *p* < 0.001.

Using the XianTao online platform, we analyzed liver hepatocellular carcinoma (LIHC) tissue data from the TCGA and GTEx databases. PSPH expression was found to be markedly elevated in LIHC samples (normal, n = 160; tumor, n = 371) (Figure 1A), a pattern that was recapitulated in paired tumor and adjacent normal tissues from 50 TCGA-LIHC patients (Figure 1B). Retrieval of 1000 PSPH-associated differentially expressed genes via Linkedomics followed by KEGG/GO enrichment analysis using DAVID revealed that the top enriched pathways included cell cycle, cellular senescence, and oocyte meiosis, and suggested potential links to hepatitis B and VEGF signaling (Figure 1C). Survival analysis indicated that higher PSPH expression was significantly associated with poorer prognosis (overall survival, OS, *p* = 0.014; progression-free interval, PFI, *p* = 0.047), whereas disease-specific survival (DSS) showed a similar but non-significant trend (Figure 1D–F). Cox proportional-hazards modeling of TCGA-LIHC clinical data showed that PSPH exhibited promising diagnostic performance (AUC = 0.892), providing supportive evidence for its biological relevance (Figure 1G). Furthermore, correlation analysis using the GEPIA2 database revealed positive associations between PSPH and the angiogenic markers VEGFA, VEGFR2, CD31, and HIF1α (Figure 1H–K). Immune-infiltration analysis showed inverse correlations between PSPH expression and multiple immune cell populations (Figure 1L), consistent with a role for elevated PSPH in promoting a hypoxic, immunosuppressive tumor microenvironment. Collectively, these data support PSPH as a likely promoter of angiogenesis during LIHC progression.

### 3.2. PSPH Promotes Metastasis and Angiogenesis of Hepatocellular Carcinoma In Vitro and In Vivo

**Figure 2:**
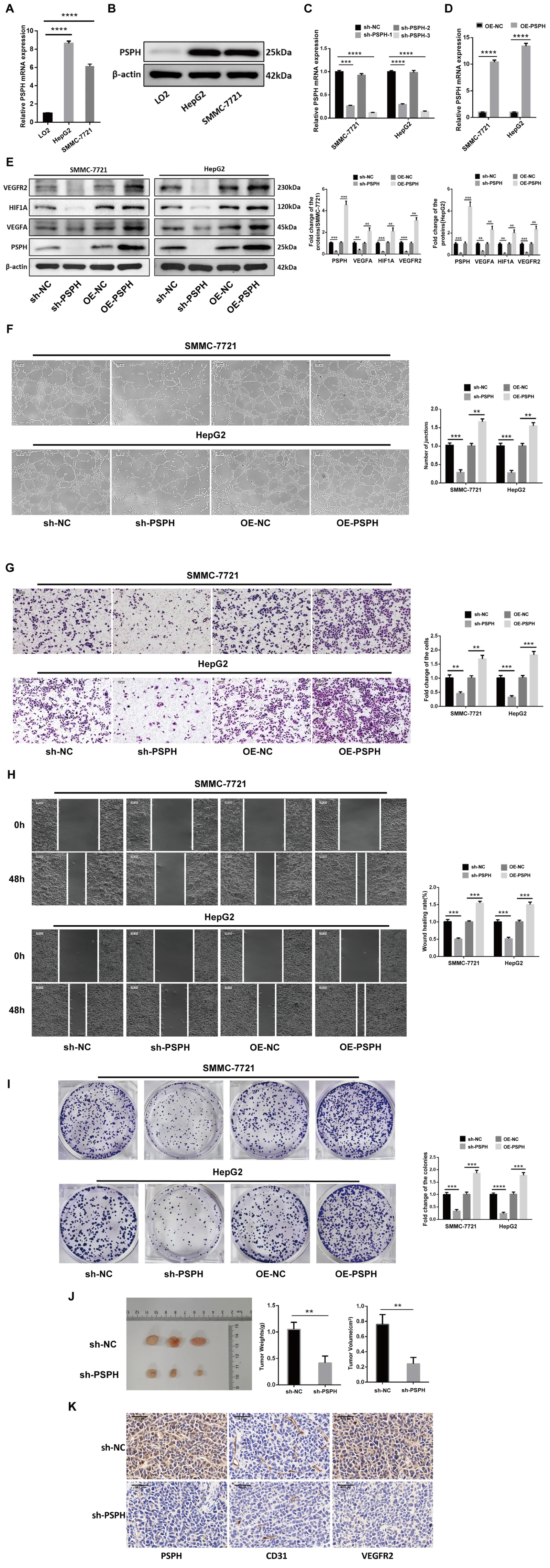
**PSPH promotes the proliferation, migration, invasion, and angiogenesis of hepatocellular carcinoma (HCC) in vitro and in vivo.** (**A**) qRT-PCR analysis of PSPH mRNA expression levels in the normal hepatic cell line LO2 and HCC cell lines (HepG2 and SMMC-7721); (**B**) Western blot analysis of PSPH protein expression levels in LO2, HepG2, and SMMC-7721 cells; (**C**) qRT-PCR verification of PSPH knockdown efficiency in SMMC-7721 and HepG2 cells; (**D**) qRT-PCR verification of PSPH overexpression efficiency in SMMC-7721 and HepG2 cells; (**E**) Western blot analysis and corresponding quantification of VEGFR2, HIF1α, VEGFA, and PSPH protein levels in SMMC-7721 and HepG2 cells following PSPH knockdown (sh-PSPH) or overexpression (OE-PSPH); (**F**) Representative images and quantification of tube-formation assays evaluating the angiogenic capacity of HUVECs cultured with conditioned media from SMMC-7721 and HepG2 cells with altered PSPH expression; (**G**) Representative images and quantification of Transwell assays evaluating the invasive capacity of SMMC-7721 and HepG2 cells following PSPH knockdown or overexpression (Scale bar = 50 μm); (**H**) Representative images and quantification of wound-healing assays (at 0 h and 48 h) evaluating the migratory capacity of SMMC-7721 and HepG2 cells following PSPH knockdown or overexpression (Scale bar = 50 μm); (**I**) Representative images and quantification of colony-formation assays evaluating the proliferative capacity of SMMC-7721 and HepG2 cells following PSPH knockdown or overexpression; (**J**) Representative images of subcutaneous tumor xenografts from nude mice, along with the statistical analysis of tumor weight and tumor volume in the sh-NC and sh-PSPH groups; (**K**) Representative immunohistochemical (IHC) staining images indicating the expression levels of PSPH, CD31, and VEGFR2 in the tumor xenograft tissues from the sh-NC and sh-PSPH groups. (Data are presented as mean ± SD. ** *p* < 0.01, *** *p* < 0.001, **** *p* < 0.0001, vs. control group.).

qRT-PCR and Western blot analyses demonstrated that PSPH expression was markedly elevated in LIHC cell lines compared with normal hepatocytes (Figure 2A,B). Stable PSPH knockdown and overexpression cell lines were established in two LIHC models, SMMC-7721 (Wuhan Walna Biology, Wuhan, China) and HepG2 (Wuhan Procell Life Science & Technology, Wuhan, China), and transduction efficiency was confirmed by qRT-PCR (Figure 2C,D). Concordant changes in the protein levels of PSPH and angiogenic markers were observed by Western blot, with VEGFA, HIF1α, and VEGFR2 varying in parallel with PSPH expression (Figure 2E). To assess the functional consequences of PSPH modulation, tube-formation, Transwell migration/invasion, wound-healing, and colony-formation assays were performed (Figure 2F–I). PSPH depletion impaired the tube-formation, motility, invasiveness, and proliferative capacities of SMMC-7721 and HepG2 cells, whereas PSPH overexpression produced the opposite effects, indicating a pro-tumorigenic role for PSPH in LIHC. In vivo, PSPH knockdown significantly inhibited subcutaneous tumor formation in nude mice, with marked reductions in tumor volume and weight (Figure 2J). Immunohistochemical analysis of the xenografts revealed decreased CD31 and VEGFR2 expression in tumors with reduced PSPH levels (Figure 2K). Taken together, these data indicate that PSPH promotes the proliferation, migration, and angiogenesis of LIHC cells both in vitro and in vivo.

### 3.3. MiR-101-3p Binds the PSPH 3′ UTR and Suppresses Angiogenesis, Invasion, and Migration of Hepatocellular Carcinoma Cells In Vitro

**Figure 3:**
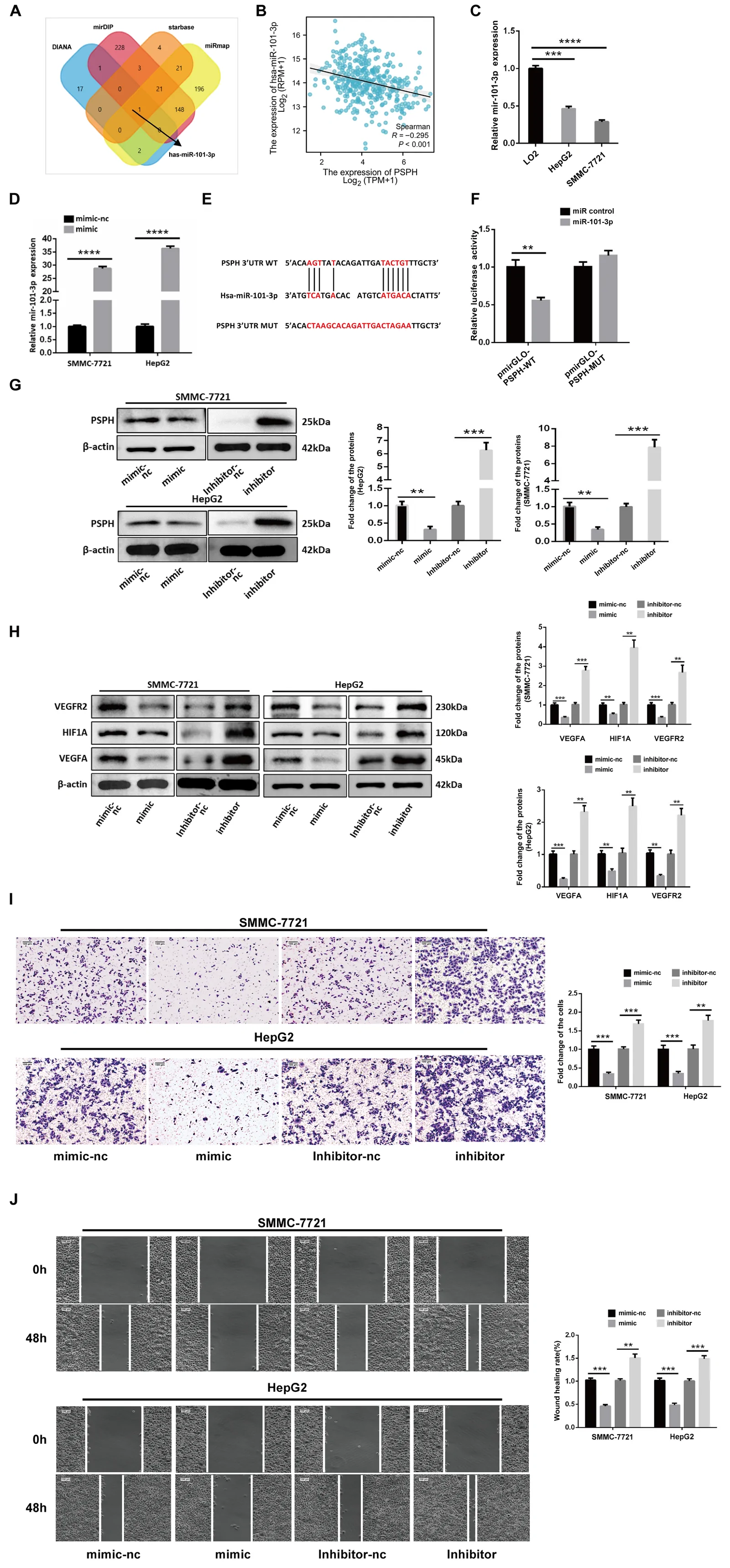
**MiR-101-3p suppresses the angiogenesis, invasion, and migration of hepatocellular carcinoma (HCC) cells in vitro by directly targeting the PSPH 3′ UTR.** (**A**) Venn diagram showing the intersection of predicted miRNAs targeting PSPH from four databases (DIANA, mirDIP, StarBase, and miRmap); (**B**) Correlation analysis between the expression levels of miR-101-3p and PSPH in HCC; (**C**) qRT-PCR analysis of miR-101-3p expression levels in the normal hepatic cell line LO2 and HCC cell lines (HepG2 and SMMC-7721); (**D**) qRT-PCR verification of miR-101-3p overexpression efficiency following mimic transfection in SMMC-7721 and HepG2 cells; (**E**) Schematic illustration of the predicted miR-101-3p binding sites within the wild-type (WT) PSPH 3′ UTR and the designed mutant (MUT) sequences; (**F**) Dual-luciferase reporter assay evaluating the relative luciferase activity of the pmirGLO-PSPH-WT and pmirGLO-PSPH-MUT vectors co-transfected with miR-101-3p mimic or mimic-nc; (**G**) Western blot analysis and corresponding quantification of PSPH protein levels in SMMC-7721 and HepG2 cells following transfection with miR-101-3p mimic or inhibitor; (**H**) Western blot analysis and corresponding quantification of VEGFR2, HIF1α, and VEGFA protein levels in SMMC-7721 and HepG2 cells following transfection with miR-101-3p mimic or inhibitor; (**I**) Representative images and quantification of Transwell assays evaluating the invasive capacity of SMMC-7721 and HepG2 cells following miR-101-3p mimic or inhibitor transfection (Scale bar = 100 μm); (**J**) Representative images and quantification of wound-healing assays (at 0 h and 48 h) evaluating the migratory capacity of SMMC-7721 and HepG2 cells following miR-101-3p mimic or inhibitor transfection (Scale bar = 100 μm). (Data are presented as mean ± SD. ** *p* < 0.01, *** *p* < 0.001, **** *p* < 0.0001, vs. control group.).

MiRNA candidates predicted to interact with PSPH were identified using DIANA, StarBase, mirDIP, and miRmap. MiR-101-3p was the only miRNA common to all four databases (Figure 3A). A significant inverse correlation between miR-101-3p and PSPH was observed in the bioinformatics analysis (*p* < 0.001) (Figure 3B). qRT-PCR revealed that miR-101-3p expression was markedly reduced in LIHC cell lines (Figure 3C), and successful overexpression was confirmed following mimic transfection (Figure 3D). To determine whether miR-101-3p regulates PSPH via direct 3’ UTR binding, wild-type and mutant PSPH 3′ UTR reporter constructs were generated. Luciferase assays demonstrated that miR-101-3p bound the PSPH 3′ UTR and suppressed reporter activity (Figure 3E,F). Consistently, Western blotting showed that miR-101-3p overexpression led to a pronounced decrease in PSPH protein levels, whereas inhibition of miR-101-3p increased PSPH expression (Figure 3G). The modulation of PSPH was accompanied by concordant changes in HIF1α, VEGFA, and VEGFR2 protein levels (Figure 3H). Functional assays indicated that miR-101-3p overexpression significantly impaired LIHC cell migration and invasion, whereas miR-101-3p inhibition produced the opposite effects (Figure 3I,J). These results indicate that miR-101-3p suppresses PSPH expression through direct binding to its 3′ UTR, thereby inhibiting angiogenic and pro-metastatic phenotypes in HCC cells.

### 3.4. MiR-101-3p Reverses PSPH-Driven Promotion of Hepatocellular Carcinoma Progression

**Figure 4:**
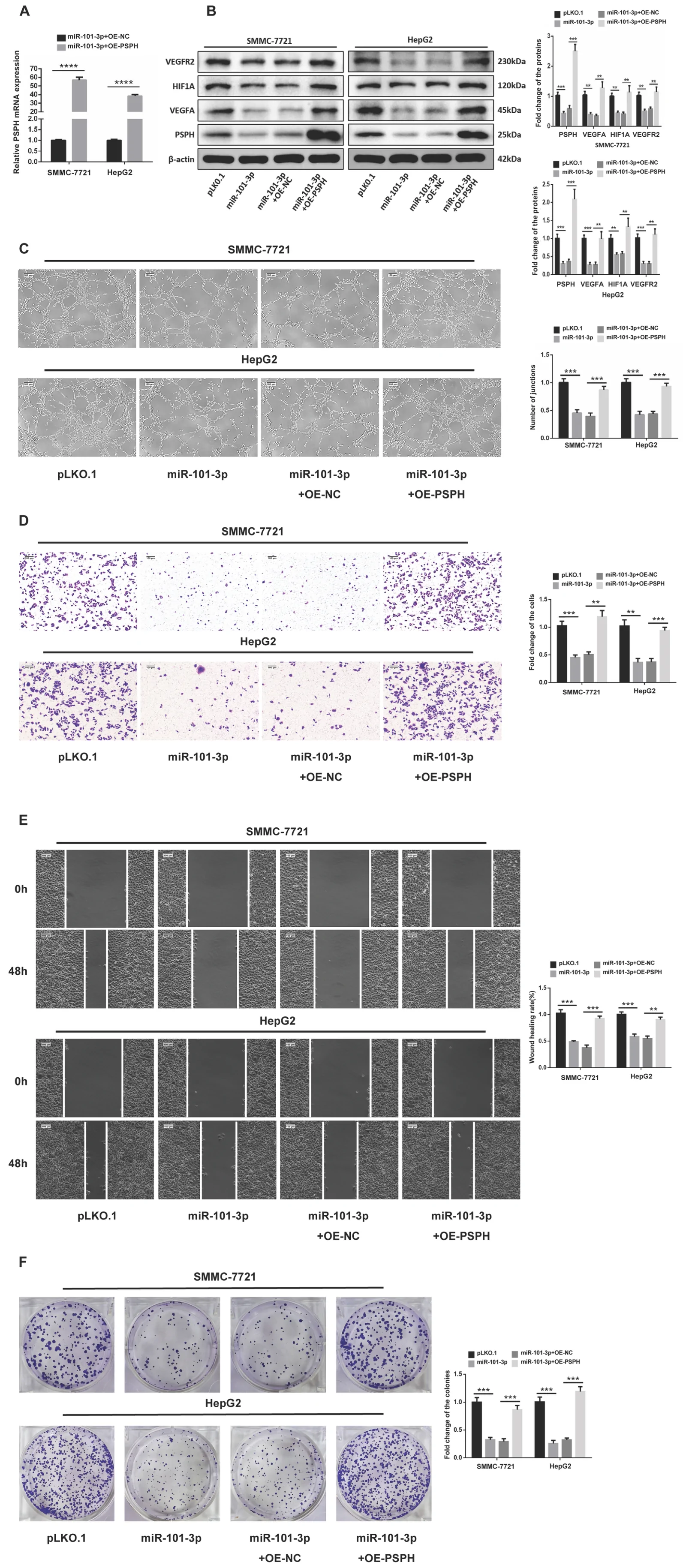
**Overexpression of PSPH reverses the tumor-suppressive effects induced by miR-101-3p in hepatocellular carcinoma (HCC) cells in vitro.** (**A**) qRT-PCR analysis of PSPH mRNA expression levels in SMMC-7721 and HepG2 cells stably expressing miR-101-3p following co-transfection with OE-NC or OE-PSPH; (**B**) Western blot analysis and corresponding quantification of VEGFR2, HIF1α, VEGFA, and PSPH protein levels in SMMC-7721 and HepG2 cells across different treatment groups (pLKO.1, miR-101-3p, miR-101-3p + OE-NC, and miR-101-3p + OE-PSPH); (**C**) Representative images and quantification of tube-formation assays evaluating the angiogenic capacity of HUVECs cultured with conditioned media from the treated SMMC-7721 and HepG2 cells (Scale bar = 100 μm); (**D**) Representative images and quantification of Transwell assays evaluating the invasive capacity of the treated SMMC-7721 and HepG2 cells (Scale bar = 100 μm); (**E**) Representative images and quantification of wound-healing assays (at 0 h and 48 h) evaluating the migratory capacity of the treated SMMC-7721 and HepG2 cells (Scale bar = 100 μm); (**F**) Representative images and quantification of colony-formation assays evaluating the proliferative capacity of the treated SMMC-7721 and HepG2 cells. (Data are presented as mean ± SD. ** *p* < 0.01, *** *p* < 0.001, **** *p* < 0.0001, vs. control group.).

qRT-PCR analysis indicated that, in LIHC cell lines stably expressing miR-101-3p, concurrent PSPH overexpression resulted in a sustained elevation of PSPH mRNA levels (Figure 4A). Consistent with this, Western blot analysis demonstrated that PSPH overexpression reversed the suppressive effects of miR-101-3p on HIF1α and angiogenic markers (Figure 4B). To determine whether PSPH could counteract the functional consequences of miR-101-3p at the cellular level, the engineered cells were subjected to tube-formation, Transwell migration/invasion, wound-healing, and colony-formation assays (Figure 4C–F). MiR-101-3p overexpression markedly inhibited LIHC cell proliferation, migration, and invasion, whereas the overexpression of PSPH effectively rescued these phenotypes. These findings indicate that PSPH overexpression can abrogate the tumor-suppressive effects of miR-101-3p.

### 3.5. LncRNA GSEC Is Upregulated in Hepatocellular Carcinoma and Mediates Post-Transcriptional Regulation via Binding to miR-101-3p

**Figure 5:**
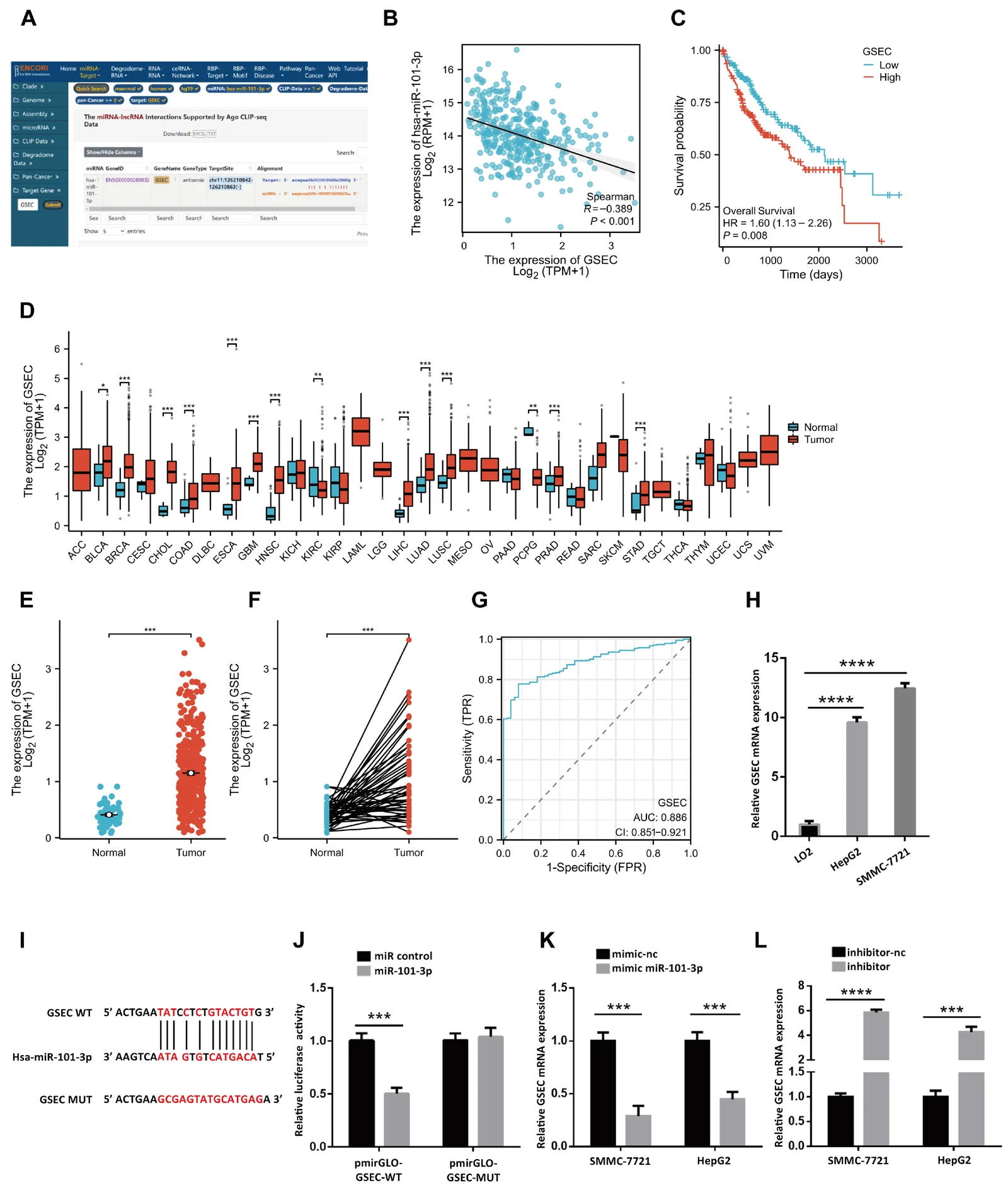
**LncRNA GSEC is upregulated in hepatocellular carcinoma (HCC) and functions as a competing endogenous RNA (ceRNA) by sponging miR-101-3p.** (**A**) Prediction of the interaction between lncRNA GSEC and miR-101-3p using the StarBase (ENCORI) database; (**B**) Spearman correlation analysis between the expression levels of GSEC and miR-101-3p in HCC; (**C**) Kaplan–Meier survival curve showing the association between GSEC expression and overall survival (OS) in HCC patients; (**D**) Pan-cancer analysis of GSEC mRNA expression levels across multiple tumor types and normal tissues based on the TCGA database; (**E**) The mRNA expression levels of GSEC in normal and HCC (LIHC) tissues from the TCGA database; (**F**) The mRNA expression levels of GSEC in paired tumor and adjacent normal tissues from TCGA-LIHC patients; (**G**) Receiver operating characteristic (ROC) curve evaluating the diagnostic value of GSEC in HCC; (**H**) qRT-PCR analysis of GSEC mRNA expression levels in the normal hepatic cell line LO2 and HCC cell lines (HepG2 and SMMC-7721); (**I**) Schematic illustration of the predicted miR-101-3p binding sites within the wild-type (WT) GSEC sequence and the designed mutant (MUT) sequence; (**J**) Dual-luciferase reporter assay evaluating the relative luciferase activity of the pmirGLO-GSEC-WT and pmirGLO-GSEC-MUT vectors co-transfected with miR-101-3p mimic or miR-control; (**K**) qRT-PCR analysis of GSEC expression in SMMC-7721 and HepG2 cells following transfection with miR-101-3p mimic or mimic-nc; (**L**) qRT-PCR analysis of GSEC expression in SMMC-7721 and HepG2 cells following transfection with miR-101-3p inhibitor or inhibitor-nc. (Data are presented as mean ± SD. * *p* < 0.05, ** *p* < 0.01, *** *p* < 0.001, **** *p* < 0.0001, vs. control group.).

StarBase analysis indicated that miR-101-3p has the potential to bind lncRNA GSEC (Figure 5A). Correlation analysis revealed an inverse relationship between miR-101-3p and GSEC (Figure 5B), and low GSEC expression was associated with improved prognosis (Figure 5C). Pan-cancer analysis of TCGA data showed GSEC overexpression across multiple tumor types (Figure 5D). In LIHC, GSEC was upregulated in 374 tumor samples compared with 50 normal liver tissues (Figure 5E), and paired analysis of 50 tumor–adjacent normal pairs confirmed elevated GSEC expression in tumor tissues (Figure 5F). Diagnostic ROC analysis yielded an AUC of 0.886, suggesting promising diagnostic potential and providing supportive evidence for the biological relevance of GSEC (Figure 5G). qRT-PCR further validated high GSEC expression in LIHC cell lines (Figure 5H). Luciferase reporter assays using wild-type and mutant GSEC binding sites confirmed that the interaction between GSEC and miR-101-3p affected reporter activity (Figure 5I,J). Following mimic and inhibitor treatments, qRT-PCR showed that GSEC expression is negatively regulated by miR-101-3p (Figure 5K,L). Collectively, these results indicate that GSEC is upregulated in LIHC tissues and cell lines, correlates with prognosis, and interacts with miR-101-3p.

### 3.6. LncRNA GSEC Affects HCC Metastasis and Angiogenesis In Vitro and In Vivo

**Figure 6:**
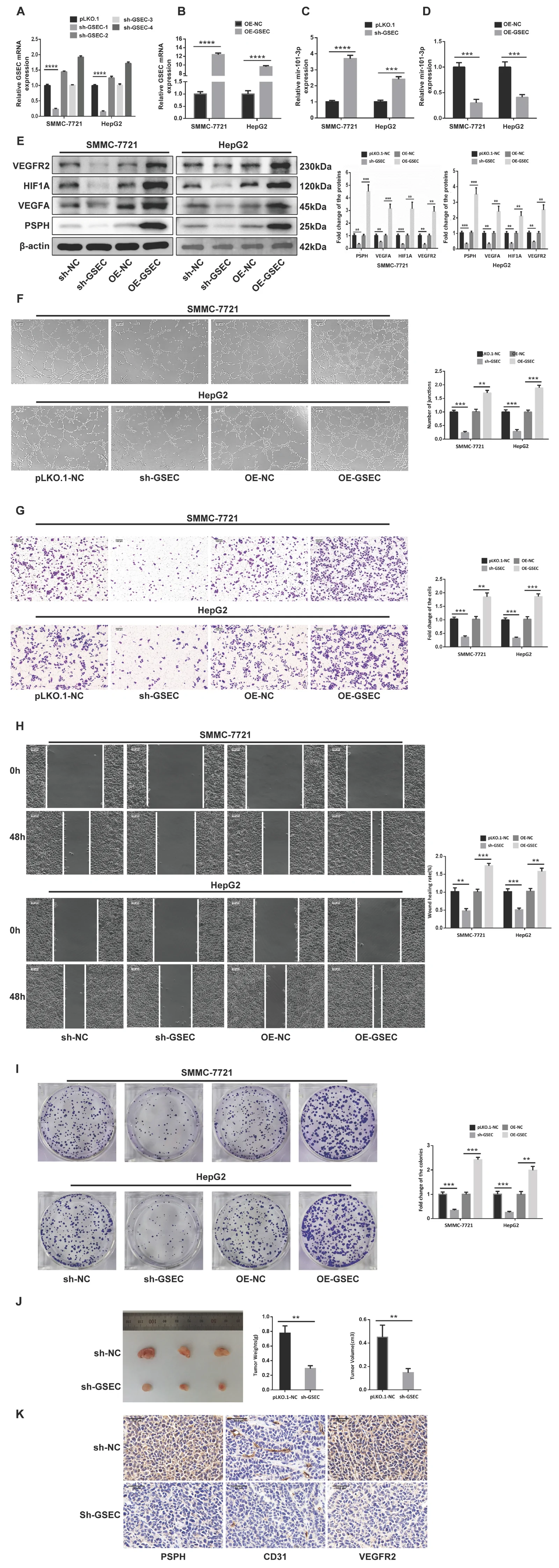
**LncRNA GSEC promotes the proliferation, migration, invasion, and angiogenesis of hepatocellular carcinoma (HCC) in vitro and tumor growth in vivo.** (**A**) qRT-PCR verification of GSEC knockdown efficiency in SMMC-7721 and HepG2 cells; (**B**) qRT-PCR verification of GSEC overexpression efficiency in SMMC-7721 and HepG2 cells; (**C**) qRT-PCR analysis of miR-101-3p expression levels in SMMC-7721 and HepG2 cells following GSEC knockdown; (**D**) qRT-PCR analysis of miR-101-3p expression levels in SMMC-7721 and HepG2 cells following GSEC overexpression; (**E**) Western blot analysis and corresponding quantification of VEGFR2, HIF1α, VEGFA, and PSPH protein levels in SMMC-7721 and HepG2 cells following GSEC knockdown (sh-GSEC) or overexpression (OE-GSEC); (**F**) Representative images and quantification of tube-formation assays evaluating the angiogenic capacity of HUVECs cultured with conditioned media from SMMC-7721 and HepG2 cells with altered GSEC expression (Scale bar = 100 μm); (**G**) Representative images and quantification of Transwell assays evaluating the invasive capacity of SMMC-7721 and HepG2 cells following GSEC knockdown or overexpression (Scale bar = 100 μm); (**H**) Representative images and quantification of wound-healing assays (at 0 h and 48 h) evaluating the migratory capacity of SMMC-7721 and HepG2 cells following GSEC knockdown or overexpression (Scale bar = 100 μm); (**I**) Representative images and quantification of colony-formation assays evaluating the proliferative capacity of SMMC-7721 and HepG2 cells following GSEC knockdown or overexpression; (**J**) Representative images of subcutaneous tumor xenografts from nude mice, along with the statistical analysis of tumor weight and tumor volume in the control (sh-NC) and sh-GSEC groups; (**K**) Representative immunohistochemical (IHC) staining images indicating the expression levels of PSPH, CD31, and VEGFR2 in the tumor xenograft tissues from the sh-NC and sh-GSEC groups (Scale bar = 50 μm). (Data are presented as mean ± SD. ** *p* < 0.01, *** *p* < 0.001, **** *p* < 0.0001, vs. control group.).

To assess the role of GSEC in HCC, stable GSEC knockdown and overexpression cell lines were established, and transduction efficiency was confirmed by qRT-PCR (Figure 6A,B). MiR-101-3p expression was subsequently measured in these engineered cell lines, further confirming their negative correlation (Figure 6C,D). Western blot analysis revealed that GSEC knockdown suppressed, whereas GSEC overexpression enhanced, the expression of HIF1α and angiogenesis-related markers (Figure 6E). In vitro functional assays demonstrated that GSEC depletion markedly inhibited LIHC cell angiogenesis, migration, invasion, and proliferation, while GSEC overexpression produced the opposite effects (Figure 6F–I). In a subcutaneous nude mouse xenograft model, GSEC downregulation significantly inhibited tumor growth, with a pronounced reduction in tumor size and volume (Figure 6J). Immunohistochemical analysis of the xenografts showed that GSEC knockdown substantially decreased tumor vascularization and reduced VEGFR2 expression (Figure 6K). Collectively, these data indicate that GSEC promotes HCC growth and metastatic potential, at least in part, by enhancing tumor angiogenesis.

### 3.7. LncRNA GSEC/miR-101-3p Promote HCC Progression via Regulation of PSPH

**Figure 7:**
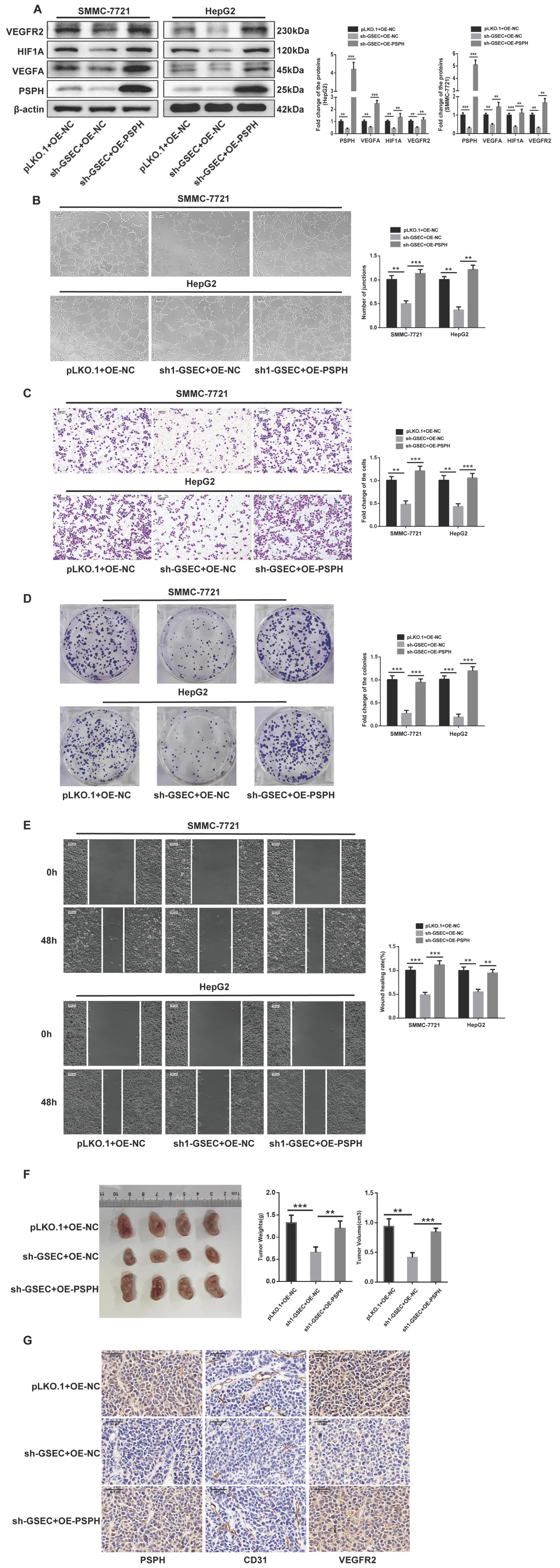
**Overexpression of PSPH reverses the tumor-suppressive effects induced by lncRNA GSEC knockdown in hepatocellular carcinoma (HCC) in vitro and in vivo.** (**A**) Western blot analysis and corresponding quantification of VEGFR2, HIF1α, VEGFA, and PSPH protein levels in SMMC-7721 and HepG2 cells across different treatment groups (pLKO.1 + OE-NC, sh-GSEC + OE-NC, and sh-GSEC + OE-PSPH); (**B**) Representative images and quantification of tube-formation assays evaluating the angiogenic capacity of HUVECs cultured with conditioned media from the treated SMMC-7721 and HepG2 cells; (**C**) Representative images and quantification of Transwell assays evaluating the invasive capacity of the treated SMMC-7721 and HepG2 cells (Scale bar = 100 μm); (**D**) Representative images and quantification of colony-formation assays evaluating the proliferative capacity of the treated SMMC-7721 and HepG2 cells (Scale bar = 100 μm); (**E**) Representative images and quantification of wound-healing assays (at 0 h and 48 h) evaluating the migratory capacity of the treated SMMC-7721 and HepG2 cells (Scale bar = 100 μm); (**F**) Representative images of subcutaneous tumor xenografts from nude mice, along with the statistical analysis of tumor weight and tumor volume across the corresponding treatment groups; (**G**) Representative immunohistochemical (IHC) staining images indicating the expression levels of PSPH, CD31, and VEGFR2 in the tumor xenograft tissues from the respective groups (Scale bar = 50 μm). (Data are presented as mean ± SD. ** *p* < 0.01, *** *p* < 0.001 vs. control group.).

Western blot analysis demonstrated that, in cell lines with stable GSEC knockdown, treatment with a miR-101-3p inhibitor further enhanced hypoxic responses, increased the expression of angiogenic markers, and restored PSPH levels that had been suppressed by sh-GSEC (Figure S1A). These findings were corroborated by Transwell and wound-healing assays, which showed that the addition of the miR-101-3p inhibitor progressively augmented the migration and invasion of HCC cell lines (Figure S1B,C). Next, sh-GSEC and OE-PSPH co-transfected HCC cell lines were generated, and PSPH expression was confirmed (Figure S1D). Western blot validation indicated that the downregulation of PSPH and VEGF pathway proteins induced by GSEC knockdown was rescued upon PSPH overexpression (Figure 7A). At the cellular level, PSPH overexpression reversed the inhibitory effects of sh-GSEC on HCC cell angiogenesis, migration, invasion, and proliferation (Figure 7B–E). In vivo, PSPH overexpression abrogated the growth suppression caused by GSEC knockdown in xenograft models (Figure 7F). Immunohistochemical analysis further showed that PSPH overexpression reversed the reduction in tumor vascularization induced by GSEC knockdown in nude mouse grafts (Figure 7G). Taken together, these results suggest that the pro-tumorigenic role of PSPH in HCC is, at least in part, driven by the lncRNA GSEC/miR-101-3p regulatory axis.

## 4. Discussion

Hepatocellular carcinoma (HCC) develops within a profoundly hypoxic microenvironment that activates HIF1α-dependent metabolic and angiogenic programs. In this study, we identify phosphoserine phosphatase (PSPH) as a previously underappreciated metabolic effector that contributes to HCC progression. Although PSPH has been implicated in metabolic adaptation and tumorigenesis in other malignancies, its role in HIF1α-dependent angiogenesis in HCC has not been defined. Our findings therefore provide new mechanistic insight into how serine metabolism intersects with hypoxia-responsive pathways in liver cancer.

We demonstrate that PSPH is markedly upregulated in HCC tissues and cell lines, correlates with angiogenic markers, and promotes proliferation, migration, invasion, and tube formation. Importantly, PSPH overexpression enhances HIF1α and its downstream angiogenic targets, suggesting that PSPH may amplify hypoxia-responsive signaling. Together with previous evidence that HIF1α can transcriptionally induce PSPH [55], these results support the existence of a PSPH–HIF1α feed-forward loop that may reinforce hypoxia signaling. While our data strongly suggest a functional connection between PSPH and HIF1α activation, the precise biochemical mechanism—such as whether PSPH directly modulates HIF1α stability or transcriptional activity—remains to be clarified. Future studies incorporating PSPH enzymatic assays and controlled hypoxia-induction experiments will be essential to determine whether PSPH acts as a bona fide hypoxia-responsive metabolic effector.

**Figure 8:**
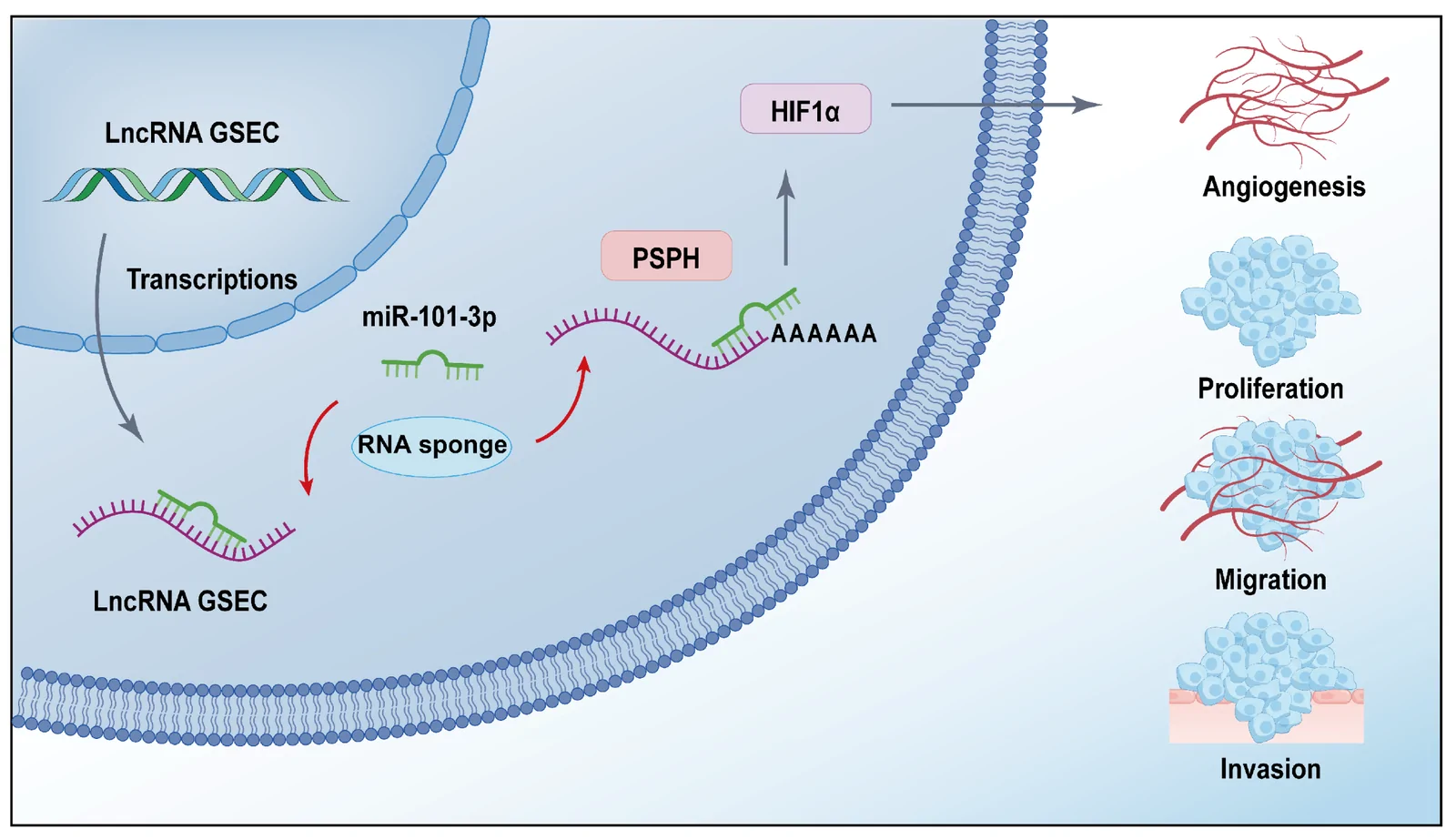
**Schematic illustration of the lncRNA GSEC/miR-101-3p/PSPH axis in HCC progression.** LncRNA GSEC acts as a competing endogenous RNA (ceRNA) to sponge miR-101-3p, thereby alleviating its suppressive effect on PSPH. The elevated PSPH subsequently activates HIF1α signaling, ultimately leading to enhanced angiogenesis, proliferation, migration, and invasion of HCC cells.

Upstream, we reveal a novel noncoding RNA regulatory axis in which lncRNA GSEC sponges miR-101-3p to derepress PSPH (Figure 8). Although miR-101-3p is a well-established tumor-suppressive miRNA in HCC, no previous studies have linked it to PSPH regulation. Likewise, the involvement of GSEC in metabolic–hypoxic signaling has not been explored. Our results therefore uncover a previously uncharacterized GSEC/miR-101-3p/PSPH axis that modulates HIF1α-dependent angiogenesis. Gain- and loss-of-function experiments, luciferase assays, and xenograft models consistently support this regulatory pathway, highlighting its biological relevance. Nevertheless, additional biochemical validation will further strengthen the mechanistic conclusions.

Beyond angiogenesis, PSPH may also influence immune modulation. PSPH downregulation has been reported to potentiate antitumor immunity and enhance responsiveness to immune checkpoint blockade [56], suggesting that PSPH-driven metabolic reprogramming may contribute to an immunosuppressive microenvironment. Moreover, hypoxia is known to impair immune effector function and promote resistance to PD-1 blockade [57], whereas alleviating hypoxia sensitizes tumors to PD-1/PD-L1 therapy [58]. Hypoxia can also activate alternative suppressive pathways to bypass anti-PD-1/PD-L1 treatment [59]. Although our bioinformatic analyses revealed negative correlations between PSPH expression and multiple immune cell populations, our experiments did not directly assess immune function; thus, the potential involvement of PSPH in immune escape should be considered a hypothesis rather than a definitive conclusion. Future studies incorporating immunological assays and tumor–immune interaction models will be required to clarify this potential role.

This study has several limitations that should be considered when interpreting the results. First, the diagnostic analyses were based on public datasets (TCGA and GTEx), and therefore required validation in independent clinical cohorts to determine their real-world applicability. Second, the mechanistic experiments were performed in established hepatocellular carcinoma cell lines, which may not fully recapitulate the heterogeneity and complexity of primary tumors. Third, although xenograft models provide valuable in vivo evidence, they lack an intact immune system, limiting the ability to evaluate immune-related mechanisms. Future studies incorporating clinical samples, patient-derived models, and immunocompetent systems will be essential to further substantiate these findings.

In summary, our study reveals a previously unrecognized metabolic–hypoxic regulatory circuit in HCC in which GSEC-mediated suppression of miR-101-3p elevates PSPH, thereby amplifying HIF1α signaling to promote angiogenesis, metastasis, and potentially immune modulation. Although several mechanistic aspects require further validation—including PSPH enzymatic activity, hypoxia-induction experiments, and clinical validation of its diagnostic performance—our findings highlight PSPH as a promising biomarker and therapeutic target and provide a foundation for future mechanistic and translational investigations.

## Data Availability

The datasets used or analyzed during the current study are available from the corresponding authors on reasonable request.

## Supplementary Materials

The following supporting information can be downloaded at https://www.mdpi.com/article/10.3390/cimb48080784/s1.
