# Supplementary material for: PSPH Promotes Hepatocellular Carcinoma Progression by Upregulating HIF-1α and Is Regulated by LncRNA GSEC/miR-101-3p
Source: Curr Issues Mol Biol. 2026 Jul 31;48(8):784. doi: 10.3390/cimb48080784 (PMC13511225; doi:10.3390/cimb48080784)
Supplement: Supplementary file 1 [file cimb-48-00784-s001.zip › supplement-figs1.pdf]

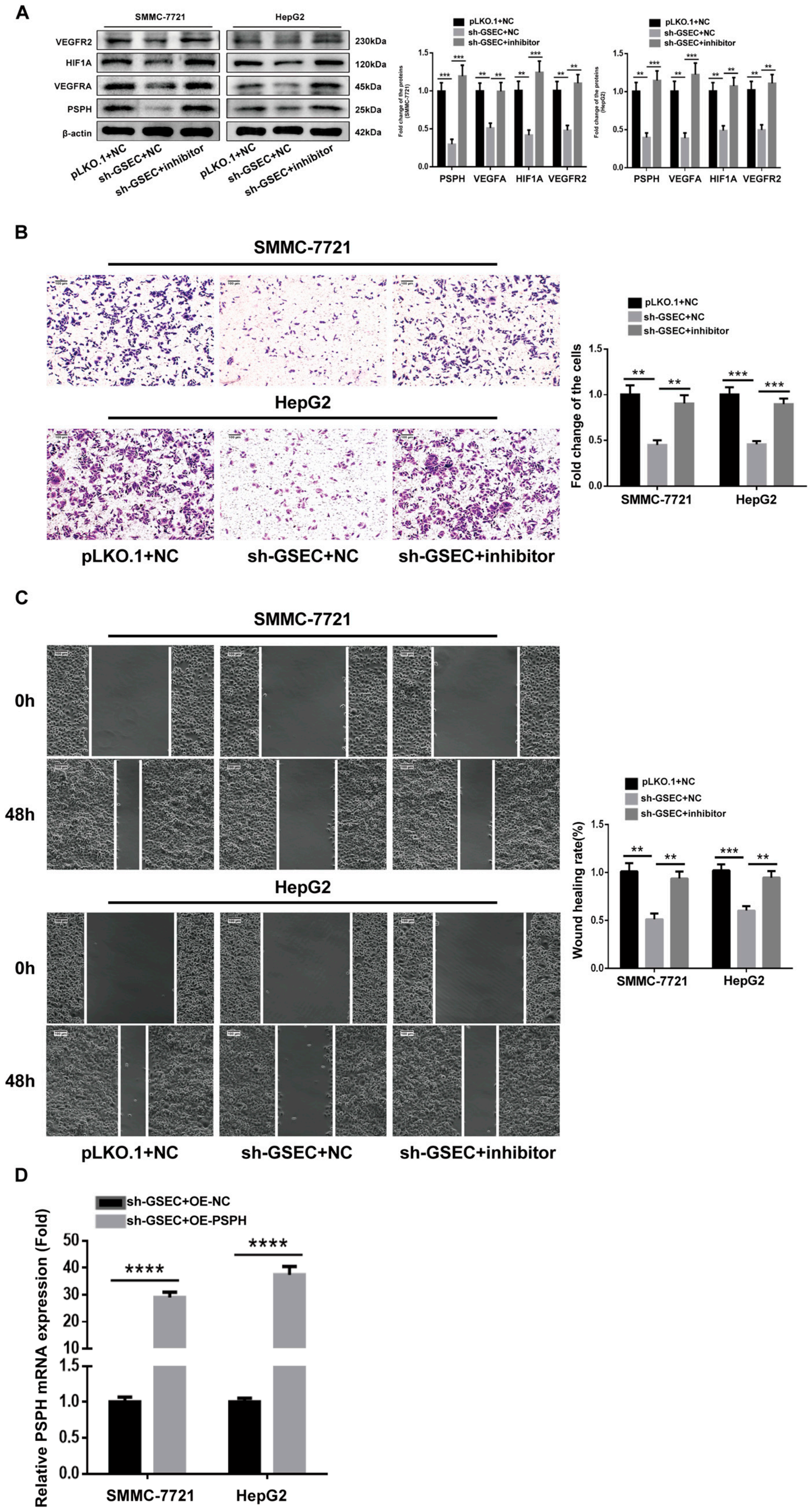

Figure S1. Rescue experiments confirming the GSEC/miR-101-3p/PSPH axis in HCC cells. (**A**) Western blot analysis of VEGFR2, HIF1A, VEGFA, and PSPH protein levels in SMMC-7721 and HepG2 cells with stable GSEC knockdown following treatment with miR-101-3p inhibitor or negative control (NC). Right panels show quantitative analysis of relative protein expression. (**B**) Representative images and quantification of Transwell

migration and invasion assays in SMMC-7721 and HepG2 cells under the indicated treatments(Scale bar = 100 μm). (**C**) Representative images and quantification of wound-healing assays at 0 h and 48 h in SMMC-7721 and HepG2 cells(Scale bar = 100 μm). (**D**) Relative PSPH mRNA expression in sh-GSEC and OE-PSPH co-transfected HCC cell lines, as determined by qRT-PCR. Data are presented as mean ± SD. *$P < 0.05$, **$P < 0.01$, ***$P < 0.001$, ****$P < 0.0001$
