# Supplementary material for: PSPH Promotes Hepatocellular Carcinoma Progression by Upregulating HIF-1α and Is Regulated by LncRNA GSEC/miR-101-3p
Source: Curr Issues Mol Biol. 2026 Jul 31;48(8):784. doi: 10.3390/cimb48080784 (PMC13511225; doi:10.3390/cimb48080784)
Supplement: Supplementary file 1 [file cimb-48-00784-s001.zip › supplement-tables.pdf]

Supplementary Table S1. Primers used for cloning and vector construction

Name	Sequence(5’-3’)
**sh1-GSEC**	CCGGGGAGGTCACAACAGTACAACTCGAGTTGTACTGTTGTGACCTCCTTTTTT
**sh2-GSEC**	CCGGGTACAAAGAATCAAAGTCACTCGAGTGACTTTGATTCTTTGTACTTTTTT
**sh3-GSEC**	CCGGCTACCAGATTCCTTGTGAACTCGAGTTCACAAGGAATCTGGTAGTTTTTT
**sh4-GSEC**	CCGGGGACTTAGGAGTCCAAAGACTCGAGTCTTTGGACTCCTAAGTCCTTTTTT
**sh1-PSPH**	CCGG-AGTCGCCTACAGGAGCGAAAT-CTCGAG-ATTTCGCTCCTGTAGGCGACT-TTTTTG
**sh2-PSPH**	CCGG-ATTCTACTTTAACGGTGAATA-CTCGAG-TATTCACCGTTAAAGTAGAAT-TTTTTG
**sh3-PSPH**	CCGG-TCTGGTGGCTTTAGGAGTATT-CTCGAG-AATACTCCTAAAGCCACCAGA-TTTTTG
**sh4-PSPH**	CCGG-TGGAGATGGTGCCACAGATAT-CTCGAG-ATATCTGTGGCACCATCTCCA-TTTTTG
**miR-101-3p**	ACCGGTGCCCTGGCTCAGTTATCACAGTGCTGATGCTGTCTATTCTAAAGGTACAGTACTGTGATAACTGAAGGATGGCAGAATTC
**PLVX-PSPH**	F: GATCTATTTCCGGTGAATTCATGGTCTCCCACTCAGAGCT
	R: GGCGGGATCCTTATTCTTCCAGTTCTCCCAGCAGC
**pmirGLO-PSPH-3'UTR-WT**	F: TGTTTAAACGAGCTCGCTAGCCATCCATTGTCGTACAGCTC
	R: GACTCTAGACTCGAGGCTAGCCCACAAATCCGTTCTGACAT
**pmirGLO-PSPH-3'UTR-MUT**	F: AATTTTTACACTAAGCACAGATTGACTAGAATTGCTTACAGTTGCCTATTACAACTTGCTATAGAAAG
	R: CTGTAAGCAATTCTAGTCAATCTGTGCTTAGTGTAAAAATTCATCTGAAGTTGTTTGGAGCTGTACGA
**PLVX-GSEC**	F: GATCTATTTCCGGTGAATTCATACCTTCCTGCTGTCCTTC
	R: GAGGGAGAGGGGCGGGATCCGCCTCCTTAGAGGTTATAACATCC
**pmirGLO-GSEC--WT**	F: TGTTTAAACGAGCTCGCTAGCATACCTTCCTGCTGTCCTTC
	R: GACTCTAGACTCGAGGCTAGCGCCTCCTTAGAGGTTATAACATCC
**PmirGLO-GSEC--MUT**	F: GCATACTTTCACTGAACGCTCATACGTACTCTTGCATTTTTG
	R: CAAAAATGCAAGAGTACGTATGAGCGTTCAGTGAAAGTATGC

Supplementary Table S2. qRT-PCR primers and miRNA mimic/inhibitor sequences.

Name	Sequence(5’-3’)
β-ACTIN	F: CCTTCCTGGGCATGGAGTC
	R: TGATCTTCATTGTGCTGGGTG
PSPH	F: CATCCATTGTCGTACAGCTC
	R: CCACAAATCCGTTCTGACAT
GSEC	F: GAGTTCATTTGCTCTCTCTGGCAC
	R: AAGAGGAGGCCTGATGGGGATA
U6	F: CTCGCTTCGGCAGCACA
	R: AACGCTTCACGAATTTGCGT
miR-101-3p	F: GGCCGCCATACAGTACTGTGA<br>R: AGTGCAGGGTCCGAGGTATTC
	RT: GTCGTATCCAGTGCAGGGTCCGAGGTATTCGCACT GGATACGACTTCAGTTA
miR-101-3p mimic NC	UCUGUACUACACAAAAGUACU
miR-101-3p mimics	UACAGUACUGUGAUAACUGAA
miR-101-3p inhibitor NC	CAGCACUUUUGUGUAGUACAA
miR-101-3p inhibitor	UUCAGUUAUCACAGUACUGUA
